# High neutrophil-to-lymphocyte ratio predicts acute allograft rejection in kidney transplantation: a retrospective study

**DOI:** 10.3906/sag-1811-41

**Published:** 2019-04-18

**Authors:** Giray ERGİN, Müge S. DEĞER, Burak KÖPRÜ, Ülver DERİCİ, Turgay ARINSOY

**Affiliations:** 1 Department of Urology, Faculty of Medicine, Yüksek İhtisas University Ankara Turkey; 2 Department of Nephrology, Faculty of Medicine, Yüksek İhtisas University, Ankara Turkey; 3 Department of Nephrology, Faculty of Medicine, Gazi University, Ankara Turkey

**Keywords:** Kidney transplantation, acute rejection, neutrophil-to-lymphocyte ratio

## Abstract

**Background/aim:**

Our research focused on the identification of easily available and sensitive markers for early prediction of acute kidney allograft rejection (AR). We aimed to investigate the association between neutrophil-to-lymphocyte ratio (NLR) and AR in kidney transplant patients.

**Materials and methods:**

The medical records of 51 kidney transplant patients [12 female/39 male; median age of 32 (IQR: 24–44) years] were evaluated retrospectively. We considered a cut-off value of >2.5 as high NLR.

**Results:**

A total of 22 biopsy-proven AR patients and 29 controls were evaluated. The AR group had a higher NLR compared to the controls (P < 0.001). NLR levels over 2.5 [95% CI: 54.88 (9.96–302.3), P < 0.001] were significantly associated with AR in univariate analysis. The NLR levels were the only significant factor associated with AR in multivariate models, in model 1 (adjusted by age and sex) [95% CI: 114 (11.1–1175), P < 0.001], and in model 2 (adjusted by steroid dosage, uric acid, and NLR) [95% CI: 4.60 (1.59–29.3), P = 0.004].

**Conclusion:**

Our data showed that higher NLR values (>2.5) are associated with AR in kidney transplant patients, leading to the conclusion that NLR might be an easily available and useful marker option for detection of AR in this patient population.

## 1. Introduction

Kidney transplantation is the best therapeutic option for end-stage renal disease (ESRD) patients. Health-related quality of life and patient survival are assumed to improve with kidney transplantation compared to conventional hemodialysis (1,2). Acute kidney allograft rejection (AR) is the most important risk factor for graft loss after transplantation, and some kidneys do not recover function even with intense antirejection therapy. Many other variables contribute significantly to the chances of rejection of kidney transplants, such as donor source, age, sex, creatinine level, blood group, Rh type, waiting time, duration of hospitalization, and vascular complications (3,4). The use of a large array of immunosuppressant drugs reduced the incidence of AR episodes, but long-term graft survival is still insufficient in this patient population. 

The neutrophil-to-lymphocyte ratio (NLR) was first described in 1967 (5) and was reported as an easy metric to assess inflammatory state. There are several studies that give evidence for its efficacy as a predictor of prognosis and/or mortality in various inflammatory states, such as cancer (6,7), acute coronary syndrome (8–10), and infections and postoperative complications (11–13). Numerous previous studies have demonstrated that an increase in NLR is associated with delayed and poor kidney graft function (14–16). 

In this retrospective study, we aimed to evaluate the association between NLR and acute kidney allograft rejection, which is an important inflammatory state in kidney transplant patients. 

## 2. Materials and methods

### 2.1. Study design and patients

We retrospectively investigated kidney transplant patients who were under follow-up treatment from September 1996 to January 2018 at the Yüksek İhtisas University Faculty of Medicine and Gazi University Faculty of Medicine. This study was conducted according to the Helsinki Declaration and the institutional review board of each university approved the study protocol. Inclusion criteria were receiving an ABO-compatible first-time kidney transplantation and medical chart achievability. Exclusion criteria were being under the age of 18, receiving a second transplantation, any proven history of cardiovascular disease or cancer, any diagnosis of BK nephropathy or CMV positivity, obesity, and active systemic, urinary, or local infection proven by a culture during the rejection. We also excluded patients who were not using steroids to eliminate the effect of steroids on neutrophil and lymphocyte distribution and to provide homogeneity for the population. The immunosuppression protocol used at our institutions consists of an antithymocyte globulin and methylprednisolone induction regimen, followed by maintenance therapy with mycophenolate mofetil/sodium or azathioprine, prednisolone, and a calcineurin inhibitor (tacrolimus or cyclosporine). 

Medical records were reviewed to find the data for the patients’ medical history, age, sex, and kidney biopsy pathology reports during AR. Acute rejection was defined by an increase of creatinine of 30% above baseline that was not attributable to any other causes, and AR was detected via Banff criteria by allograft biopsy in all cases (17). 

### 2.2. Laboratory data

NLR was calculated with the formula of neutrophil count divided by lymphocyte count. We used blood drawn on the day of the application to the clinic for the acute rejection group and on the last outpatient clinic visit day for the control group. Different values of NLR are used in the literature in different disease states. Currently there is no universally accepted reference value, but a ratio above 3 is accepted as a high value in some disease states. In our study, serial receiver operating characteristic (ROC) curve analysis of NLRs revealed the 75th quartile (>2.5) to be the most sensitive and specific determinant of AR, leading us to consider values of 2.5 or greater as elevated. We used age-matched kidney transplant patients who had no rejection episode as a control group. We also retrospectively obtained serum biochemistry results, uric acid levels, and erythrocyte sedimentation rates. All immunosuppressive regimens were noted. 

### 2.3. Statistical analysis

All collected data were analyzed with SPSS 24.0 for Mac (IBM Corp., Armonk, NY, USA). P < 0.05 was considered statistically significant. All comparison analyses were performed by nonparametric tests due to restricted sample size. Characteristics were compared using the Wilcoxon rank sum test for baseline continuous variables and the chic-square test for categorical variables. Spearman’s test was used for the detection of correlation between variables. The ROC curve was calculated and the area under the curve (AUC) was analyzed for determination of the cut-off value of NLR. Results were expressed as median and interquartile range (IQR). Binary logistic regression analysis was used to investigate factors affecting AR. Because of the limitation of our sample size and to avoid overfitting, 3 independent variables were included in regression analyses in multivariate models. 

## 3. Results

We included the results of 51 kidney transplant patients who matched our inclusion/exclusion criteria in our analysis. Of those patients, 9 received deceased and 42 received living donor kidney transplantation. All patients in the study groups received a standardized immunosuppressive regimen consisting of calcineurin inhibitors and mycophenolate mofetil/sodium or azathioprine and steroids. Acute allograft rejection occurred in 22 of those subjects (41%). 21 experienced acute cellular rejection, and 1 experienced antibody-mediated rejection. The median time in the AR group between renal transplantation and acute rejection was 7.5 (0.25–193) months. The median time of required follow-up was 8 (3–25) months for the AR group and 25 (8–60) months for the control group. Characteristics of the study population are provided in Table 1.

**Table 1:** Characteristics of complete study population.

Variables	n = 51
Age, years, median (IQR)	32 (24–44)
Sex, male / female	39 / 12 (76.5% / 23.5%)
Donor type, deceased / living	9 / 42 ( 17.6% / 82.4%)
Median time of follow-up, months, median (IQR)	14 (4–48)
Diabetes, negative / positive	50 / 1 (98% / 2%)
Hypertension, negative / positive	44 / 7 (86.3% / 13.7%)
Serum creatinine, mg/dL, median, (IQR)	1.2 (1–1.4)
White blood cells, mm3, median (IQR)	8630 (6400–11000)
Uric acid level, mg/dL, median (IQR)	6.1 (5.2–7.4)
ESR, mm/h, median (IQR)	26.5 (12.8–44.8)
NLR, median (IQR)	1.93 (1.2–3.63)

IQR: Interquartile range, ESR: erythrocyte sedimentation rate, NLR: neutrophil-tolymphocyte ratio.

Comparisons of the demographics and clinical features between the AR and control groups are summarized in Table 2. The patients were similar in terms of age, sex, and laboratory assessments of baseline creatinine, white blood cell count, and erythrocyte sedimentation rates. The median serum uric acid level was statistically significantly higher in the AR group (P = 0.043). There was 1 patient with diabetes mellitus in the control group but none in the AR group, and the control group had more hypertensive patients than the AR group.

**Table 2:** Comparison of demographical characteristics and biochemical parameters in study subgroups.

Variables	AR (n = 22)	Control (n = 29)	P-values
Age, years, median (IQR)	30 (24–38)	35 (27–47)	0.105
Sex, male / female (n)	19 / 3	20 / 9	0.192
Donor type, deceased / living (n)	1 / 21	8 / 21	0.06
Median time of follow-up, months, median (IQR)	8 (3–25)	25 (8–60)	0.05
Diabetes, negative / positive	22 / 0	28 / 1	-
Hypertension, negative / positive (n)	21 / 1	23 / 6	0.550
Creatinine, mg/dL, median (IQR)	1.2 (1.0–1.5)	1.1 (1.4–0.9)	0.307
White blood cells, mm3, median (IQR)	9700 (6857–12800)	7500 (6345–10250)	0.223
Uric acid level, mg/dL, median (IQR)	6.9 (5.4–8.1)	5.8 (4.8–6.8)	0.043
ESR, mm/h, median (IQR)	30 (24–45)	22 (11–49)	0.531
NLR, median (IQR)	4.06 (3.11–7.03)	1.24 (1.08–1.57)	<0.001

IQR: Interquartile range, ESR: erythrocyte sedimentation rate, NLR: Neutrophil-to-lymphocyte ratio.

The median NLR level was statistically significantly higher in the AR group, with a median of 4.06 (3.11–7.03) in the AR group versus a median of 1.24 (1.08–1.57) in the control group (P < 0.001) (Figure). 

**Figure 1:**
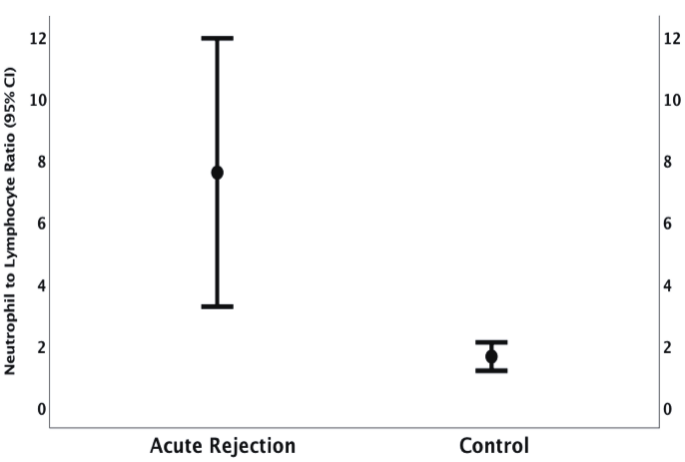
Comparison of NLR values between acute kidney rejection group and controls: the median NLR level was statistically significantly higher in AR group [4.06 (3.11–7.03) in AR group versus 1.24 (1.08–1.57) in control group (P < 0.001)].

Univariate binary logistic regression analysis was performed to analyze each factor affecting AR. Serum uric acid levels [95 % CI: 1.588 (1.014–2.486), P = 0.043] and NLR >2.5 [95% CI: 54.88 (9.96–302.3), P < 0.001] were statistically significantly associated with AR. Also, having living donor transplantation seemed to reduce the chances of rejection with marginal statistical significance [95% CI: 0.125 (0.014–1.089), P = 0.06] (Table 3). For multivariate analysis, we created 2 models, each with a maximum of 3 independent variables due to the restricted sample size. Only NLR was statistically significantly associated with AR [95% CI: 114 (11.1–1175), P < 0.001] in the first multivariate model (adjusted by age and sex). The statistically significant association between serum uric acid level and AR disappeared in this model (Table 3). The second model was adjusted by steroid dosage, serum uric acid level, and NLR. In this model, steroid dosage was included to exclude a possible effect on NLR. We also checked the association between steroid dosage and NLR to determine the confounder effect (interaction) for model 2 and found no statistically significant association between steroid dosage and NLR [95% CI: 0.96 (–2.39 to 2.49), P = 0.96]. The second multivariate model revealed that only NLR was statistically significantly associated with AR [95% CI: 4.60 (1.59–29.3), P = 0.004] (Table 3). 

**Table 3:** Determinants of acute kidney rejection (univariate and multivariate regression analysis).

Univariate model		
Variables	Βeta coefficient(confidence interval)	P-values
Age, years	1.04 (0.99–1.09)	0.118
Sex, male	0.35 (0.08–1.49)	0.157
Donor type, living	0.125 (0.014–1.089)	0.06
Baseline creatinine, mg/dL	2.526 (0.578–11.032)	0.218
ESR, mm/h	1.015 (0.976–1.056)	0.455
Uric acid, mg/dL	1.588 (1.014–2.486)	0.043
NLR, >2.5	54.88 (9.96–302.3)	<0.001
Multivariate model 1*		
Uric acid, mg/dL	1.547 (0.961–2.488)	0.072
NLR, >2.5	114 (11.1–1175)	<0.001
Multivariate model 2**		
Uric acid, mg/dL	2.563 (0.857–7.665)	0.092
Steroid dose, mg	1.294 (0.937–1.788)	0.118
NLR, >2.5	4.601 (1.593–13.293)	0.004

ESR: Erythrocyte sedimentation rate, NLR: neutrophil-to-lymphocyte ratio. *Model 1: Adjusted by age and sex. **Model 2: Mixed case model (adjusted by steroid dosage, uric acid, NLR).

## 4. Discussion

In this study, we retrospectively examined kidney transplant patients who had AR and analyzed the association with their NLR levels obtained on the day of application to the clinic. We also included age-matched control patients who had not experienced AR in our analysis and found that the NLR in the AR group was significantly higher compared to the control group. Both univariate and multivariate analyses showed that NLR of >2.5 was statistically significantly associated with AR. 

The NLR is an inflammatory marker that is inexpensive and easily available in routine clinical practice. There are several reports in the literature providing evidence that NLR is strongly associated with morbidity or mortality in several inflammatory conditions such as cancer and cardiovascular disease, as well as being a predictor for infectious and postoperative conditions (7,11,13). NLR levels were positively correlated with inflammatory cytokines in ESRD patients (18). Another study showed that kidney transplant patients had a higher NLR than healthy subjects (19) and concluded that the higher values were due to the ongoing inflammation in these patients. A prospective study by Cankaya et al. also reported that mean NLR levels were improved at the first year after transplantation but did not reach the levels found in healthy controls, which is further evidence for chronic inflammation as permanent in kidney transplantation (15). 

AR is the major cause of graft dysfunction in these patients. Some of the patients do not recover even with high-dose antirejection treatment. Most AR episodes are overlooked by both clinicians and patients, since they are mostly asymptomatic, but even if this is the case AR episodes have a negative impact on long-term graft survival. It is important to use an easily applicable marker for both the prediction and early detection of AR for this population. A rise in serum creatinine is an important and widely used laboratory test for predicting AR, but creatinine increases indicate significant histological damage in the kidney, which is a reflection of the late course of the rejection episode. 

Our results present favorable findings regarding using NLR as a simple, easily available, inexpensive marker for the assessment and prediction of AR. An NLR level above 2.5 appears to be a sensitive and specific predictior of AR, according to ROC curve analysis. Both univariate and multivariate logistic regression analysis found it independently associated with AR. We believe the increase in NLR in AR patients was unrelated to corticosteroid dosage, because all the patients were using the same maintenance dosage and our interaction analysis did not show any significant association as a confounder effect on the prediction model. 

Halazun et al. also found that elevated preoperative NLR levels were associated with a higher risk of developing delayed graft function, another state that has a shared casual mechanism with AR episodes, after kidney transplantation (16). They concluded that the preoperative active inflammatory response that was detected by higher NLR levels might affect the immediate graft function. They found that NLR over 3.5 acted as a predictor for delayed graft function. However, in our study, patients with NLR over 2.5 were more likely to develop AR. There is no universal cut-off level for NLR values; most studies have reported the levels according to the median and higher quartile. A study from a nongeriatric and healthy population reported that a normal value for NLR is between 0.78 and 3.58 (20). In other disease states such as cancer, and in cardiovascular and surgical patients, NLR values over 3.5 were accepted as a prediction value for each disease (21,22). NLR over 2.5 in heart transplantation and over 3.5 in kidney transplantation were found as predictors for RRT and graft survival in several studies (16,23). We found that the NLR cut-off value of 2.5 has 90% specificity and 60% sensitivity for detection of AR. 

There are several limitations of our study. First, the small sample size impedes generalizability of results. The retrospective design limits our interpretation of causality. A single measurement of NLR might not accurately reflect the relationship over time. Future studies that obtain serial changes of NLR would be useful to clarify to role of NLR in the follow-up of AR. 

We still believe that NLR is a practical marker in both inpatient and outpatient settings, and the accumulation of studies reporting the usefulness of practical and inexpensive markers for either diagnosis or follow-up of chronic disorders is important. The reduction of expenditure is of great of importance in health care all over the world. 

In conclusion, even with these limitations, our present study was able to detect NLR as being higher in AR patients compared to those without rejection, which might indicate an increased risk of AR associated with higher NLR levels. The findings from this retrospective study must be confirmed. Larger, prospective, controlled, and long-term follow-up studies are needed to further determine the sensitivity, specificity, and predictability of this inexpensive, convenient test in the early diagnosis of acute graft rejection. Further validation and feasibility studies are required before it can be considered for routine clinical use.

## Acknowledgment

Part of this study was presented as a poster presentation at the 30th National Congress of Nephrology, Hypertension, Dialysis, and Transplantation in Antalya, Turkey (2012).
